# Resveratrol-Loaded Chia Seed Oil-Based Nanogel as an Anti-Inflammatory in Adjuvant-Induced Arthritis

**DOI:** 10.3390/gels9020131

**Published:** 2023-02-03

**Authors:** Obaid Afzal, Abdulamalik S. A. Altamimi, Mubarak A. Alamri, Ali Altharawi, Manal A. Alossaimi, Md Sayeed Akhtar, Fauzia Tabassum, Waleed H. Almalki, Tanuja Singh

**Affiliations:** 1 Department of Pharmaceutical Chemistry, College of Pharmacy, Prince Sattam bin Abdulaziz University, Al-Kharj 11942, Saudi Arabia; 2 Department of Clinical Pharmacy, College of Pharmacy, King Khalid University, Abha 62529, Saudi Arabia; 3 Department of Pharmacology, College of Dentistry and Pharmacy, Buraydah Private Colleges, Buraydah 51418, Saudi Arabia; 4 Department of Pharmacology and Toxicology, College of Pharmacy, Umm Al-Qura University, Makkah 24382, Saudi Arabia; 5 Department of Botany, Patliputra University, Patna 800020, India

**Keywords:** resveratrol, chia seed oil, nanoemulsion, phase diagram, skin permeation, pro-inflammatory cytokines, arthritis index

## Abstract

Natural anti-inflammatory nutraceuticals may be useful in preventing rheumatoid arthritis from worsening. Resveratrol (RV) and chia seed oil, having antioxidant potential, can assist in avoiding oxidative stress-related disorders. This investigation developed and evaluated resveratrol-loaded chia seed oil-based nanoemulsion (NE) gel formulations through in vitro and in vivo studies. The physical stability and in vitro drug permeability of the chosen formulations (NE1 to NE10) were studied. The optimized RV-loaded nanoemulsion (NE2) had droplets with an average size of 37.48 nm that were homogeneous in shape and had a zeta potential of −18 mV. RV-NE2, with a permeability of 98.21 ± 4.32 µg/cm^2^/h, was gelled with 1% carbopol-940P. A 28-day anti-arthritic assessment (body weight, paw edema, and levels of pro-inflammatory mediators including TNF-α, IL-6, IL-1β, and COX-2) following topical administration of RV-NE2 gel showed significant reversal of arthritic symptoms in arthritic Wistar rats induced by Freund’s complete adjuvant injection. Therefore, RV-NE2 gel demonstrated the potential to achieve local therapeutic benefits in inflammatory arthritic conditions due to its increased topical bioavailability and balancing of pro-inflammatory mediators.

## 1. Introduction

Rheumatic arthritic conditions are a major cause of disability and locomotor dysfunction in the adult population [1]. Rheumatoid arthritis and osteoarthritis are the two most common types of these articular disorders [1]. Rheumatoid arthritis is a degenerative joint disease characterized by the slow degradation of articular cartilage. Rheumatoid arthritis is an autoimmune disorder triggered by the production of pro-inflammatory cytokines (such as TNFα, IL-6, IL-1β, and COX-2) [1]. Pharmacotherapy is used to treat different types of arthritis [1]. Traditional first-line therapies include simple analgesics and steroidal and non-steroidal anti-inflammatory drugs [2]. However, the predominant treatment has a low level of effectiveness, high rate of noncompliance from patients, and repeated, possibly severe side effects [2]. The US Food and Drug Administration (FDA) has discontinued a number of anti-inflammatory medications due to serious cardiovascular adverse effects; as a result, plant-based medications are being advocated for the treatment of arthritis [2].

The use of hydrophobic (or weakly water-soluble) drugs in topical liquid/semi-solid formulations has been established in several investigations [1,2]. Before adding hydrophobic medications to liquid or semi-solid formulations, encapsulating them in a nanoemulsion (NE) considerably improves their transport through the skin barrier [3]. In comparison to other colloidal carriers, NE as a topical carrier system is particularly advantageous since it has little skin irritation, good penetration, and a high drug-loading capacity [3]_._ The low viscosity of the NE system and difficulty of applying it necessitate its transformation into a gel for practical use. Poorly water-soluble medicines can be added to NE [3]. NE can increase a hydrophobic drug’s solubility [3,4]. The shelf life of NE is greater than that of conventional emulsions and it is a thermodynamically stable system with droplet sizes ranging from 10 to 100 nm [3]. Additionally, NE is macroscopically uniform and optically transparent [3,4]. Additionally, the preparation does not require a significant amount of energy input during manufacturing, making it easy to scale up and manufacture at a lower cost [3,4]. NE is made up of an aqueous phase, an oil, and a combination of a surfactant and co-surfactant [3,4]. It can be saturated with medications with limited water solubility and improve drug penetration via the skin, thereby increasing their bioavailability [4]. NE may also enhance medication stability by encapsulating medicinal compounds in nanodroplets [4]. A gelling agent is frequently used to improve the viscosity of NE since its high fluidity restricts it to topical applications [4]. It is expected that in these situations, topical NE-based formulations with high skin-penetration rates will perform better than conventional emulsion-based cream formulations [5].

Herbs have been used for thousands of years to treat various illnesses and even improve bodily performance [6]. Resveratrol (RV), found in herbs, has been recognized as a vital component that significantly contributes to improved overall health and reduces the risk of many illnesses [6]. As a secondary metabolite, it can be found in red wine and fruits. Its presence has also been found in numerous other plant species, legumes, peanuts, and beers [6]. Because RV is made up of two phenolic rings connected by a styrene double bond, it can produce cis- and trans-oriented 3, 40, 5-tryhydroxystilbene [6]. Grapes (*Vitis vinifera*) are commonly used to make the red wine. RV has benefited people throughout the world in numerous medical concoctions [6]. The literature shows favorable outcomes for RV in treating or preventing numerous diseases, including arthritis [6]. However, RV has limited therapeutic applications due to its weak solubility, increased metabolism, and poor absorption [7]. It also has restrictions for administration via the oral route [7]. Numerous phytoconstituents, including resveratrol (RSV) and curcumin, have been reported to have strong anti-inflammatory properties by inhibiting nuclear factor kappa B and cytokine (interleukin [IL]-1, IL-6, and tumor necrosis factor alpha) signaling. Researchers developed a transdermal gel that contained methotrexate-RSV-loaded nanoemulsions (NEs) to combat the side effects of RA monotherapy and problems with bioavailability [4]. Subsequently, the anti-inflammatory and possible anti-arthritic activities of the combination in the nanocarrier were evaluated in rats, revealing a 78.76 ± 4.16% decrease in inflammation and superior anti-arthritic effects [4]. Therefore, combining dual delivery with nanotechnology should result in effective treatment options for rheumatic disorders [4]. Recent studies have reported the synergistic antioxidant potential of chia seed oil and curcumin used in combination [8]. Another study reported that freeze-dried microencapsulated chia seed oil had better stability against lipid peroxidation and preserved the antioxidant potential due to the possession of omega-3 fatty acids [9]. Recent research led to the discovery of RV, which decreases the symptoms of inflammatory disorders but has a limited therapeutic effect due to higher metabolism and limited bioavailability [4,7]. In this context, there is tremendous interest in increasing the RV concentration along with the skin permeability of chia seed oil using NE-based gel for arthritis management. This research may synergize the therapeutic effects against arthritis due to the action RV and possession of omega 3 fatty acids in chia seed oil. Therefore, this research may lead to an effective approach for arthritis management.

## 2. Results and Discussion

### 2.1. Identification of λ_max_ for RV

The UV absorption spectrum of RV in ethanol showed a peak at 305 nm. Therefore, the significant peak at this wavelength confirmed the identification of RV, which may be ascribed to the low excitation energy of RV [10].

### 2.2. Solubility Studies for Excipient Screening

In this investigation, various oils, both natural and synthetic, were tested for RV solubility. The oil phase was chosen with the intention of incorporating as much RV as possible and making an ideal formulation for topical use. As per the solubility study, RV (120 ± 15 mg/mL) had maximum solubility in chia seed oil, which was therefore selected as the oil phase. Next, the solubilization efficiency of surfactants and co-surfactants was considered [11]. To achieve the ideal nanoemulsion, the surfactant must minimize interfacial tension, provide a flexible film that can bend around droplets, and have an acceptable hydrophilic lipophilic balance (HLB) to provide the requisite curvature at the interfacial area [11]. In most circumstances, a co-surfactant is needed to provide temporary negative interfacial tension and a fluid interfacial film [11]. Since RV was found to have maximum solubility in Cremophor^®^EL (89 ± 7 mg/mL) and Transcutol^®^P (68 ± 8 mg/mL), they were selected as the surfactant and co-surfactant, respectively.

### 2.3. Construction of Pseudo-Ternary Phase Diagrams and Formulation of RV-NE

**Figure 1:**
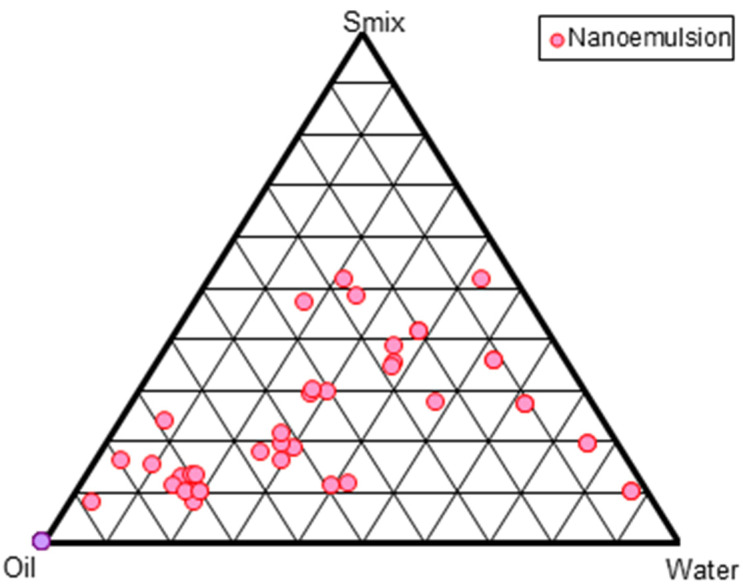
Pseudo-ternary phase diagram shown at S_mix_ (surfactant-cosurfactant) ratio of 3:1.

Several mixes of Cremophor^®^EL and Transcutol^®^P were used to construct pseudo-ternary phase diagrams. The region of the phase diagram where NE occurred was profoundly influenced by the weight ratios of the surfactant and co-surfactant. Increasing the co-surfactant concentration (S_mix_; 1:2, 1:3, 1:4) may have a destabilizing impact, as evidenced by the narrowing of the NE zone and increased frequency of NE breakdown during storage. S_mix_ ratios of 1:1 < 2:1 < 4:1 < 3:1 produced the largest NE zones (Figure 1). A high surfactant concentration decreases the interfacial barrier and provides a thermodynamically stable NE due to its tiny size, huge surface area, and charge [4]. From each S_mix_ pseudo-ternary phase diagram, multiple formulations were chosen. The thermodynamic stability experiments showed turbidity or phase separation at 1:0, 1:1, and 1:2 S_mix_ ratios, which may be attributed to the larger particle size after centrifugation. There was an opalescence that could be reversed by standing up the vial. A rise in enthalpy, caused by heating, causes chaos, whereas a decrease in entropy, caused by cooling, causes coalescence and an increase in particle size. However, the 3:1 S_mix_ ratio produced the largest NE surface area, as depicted in Figure 1. Ten formulations NE1 to NE10 were chosen at random from the pseudoternary phase diagram. Their compositions are shown in Table 1. Furthermore, this selected formulation was used for further physical stability tests.

### 2.4. Thermodynamic Stability Study

Additionally, each selected formulation was tested for thermodynamic stability [12]. The results of the heating-cooling, centrifugation, and freeze-thaw tests are displayed in Table 2, and they all revealed that initially (one week), none of the formulations displayed creaming, cracking, phase separation, or even a noticeable increase in particle size. In the second week and afterwards, the NE6-NE10 formulations did not pass the thermodynamic tests (Table 2). This may have been the result of the lower oil concentration (up to 6% oil), higher oil concentration (>11% oil), and larger particle size. Additionally, a larger S_mix_ is needed to solubilize a higher proportion of oil [12,13], while a smaller amount of oil is unable to capture a significant amount of drug. Therefore, >6% to 11% *w/w* oil for NE was the optimal range and showed physical stability. Thus, the NEs in these ranges of oil concentration were selected for further study.

### 2.5. In Vitro Drug Release Studies

**Figure 2:**
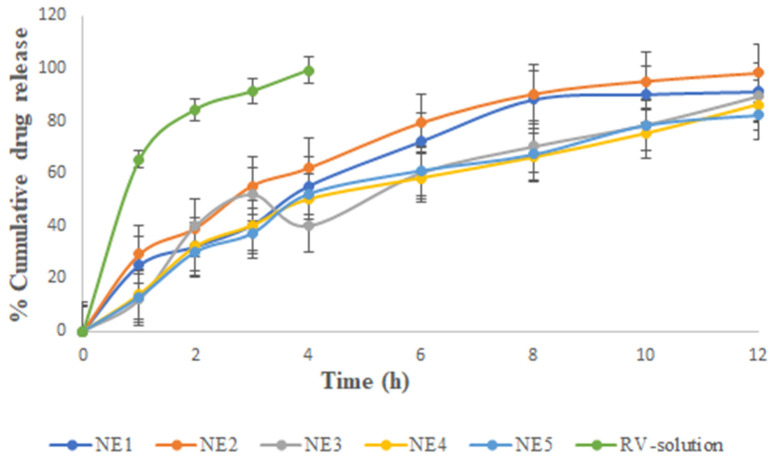
Percentage of cumulative drug release vs. time for the various selected nanoemulsions and RV solution. The average value SD (*n* = 3) is represented by each cross bar. NE: nanoemulsion.

Figure 2 illustrates that the in vitro release of RV ethanolic solution showed the highest drug release and followed NE1 to NE5. RV solution showed 65.21% drug release in vitro at 60 min, followed by 99.21% at 4 h. The cumulative release profile of RV from ethanol was faster, indicating that RV was able to move freely across the cellulose acetate membrane. Although this behavior was predicted throughout the test period, the experiment was discontinued after 4 h due to the drying of the vehicle and membrane (despite the use of occlusive circumstances, i.e., covering the donor compartment with Parafilm^®^). Moreover, this result was supported in the literature [14]. Among the NEs, the maximum drug release occurred in 12 h for NE2, as seen in the images illustrating the release patterns. As a result, RV was projected to be mostly dispersed in the oil phase of the NE. This reduced the RV release rate from the oil core, and therefore decreased its passage through the membrane over the RV solution. Among the NEs, the amount of drug that permeated from different NEs over the course of 12 h was observed in the following order: NE5 (82.21 ± 4.12%) < NE4 (86.12 ± 3.78%) < NE3 (89.31 ± 5.12%) < NE1 (91.14 ± 3.21%) < NE2 (98.21 ± 3.32%). The drug permeation flux was observed to flow in the order of: NE2 (2.21 ± 0.031 µg cm^−2^h^−1^) > NE1 (1.95 ± 0.024 µg cm^−2^ h^−1^) > NE3 (1.30 ± 0.04 µg cm^−2^ h^−1^) > NE4 (1.05 ± 0.03 µg cm^−2^ h^−1^) > NE5 (0.81 ± 0.03 µg cm^−2^ h^−1^). It was observed that the NE2 formulation had the optimal quantity of oil, regardless of the surfactant concentration, while the NE5 formulation, which was comparable, had the lowest permeation and a relatively low-permeation flux value. NE1 had the second largest permeation flow after NE2. There were subtle differences in the permeation profiles due to differences in composition and oil content (8% in NE2 versus 9% in NE1), as shown in Table 1.

From the results, it could be concluded that the surfactant concentration had a smaller effect on the drug permeation profile and flux than the S_mix_ value. We observed the surprising coexistence of two seemingly contradictory findings in this blend. First, NE1 had higher S_mix_ (51%) than NE2 (45%). It was observed that the amount of chia seed oil and RV had the least influential factor in improving drug permeation, whereas the combined action of the surfactant and cosurfactant had the largest influence. Evidence from prior studies showed that the amount and type of surfactant significantly affected the skin’s ability to absorb drugs [11,13,14,15,16].

### 2.6. Characterization of the Optimized Formulation NE (NE-2)

**Figure 3:**
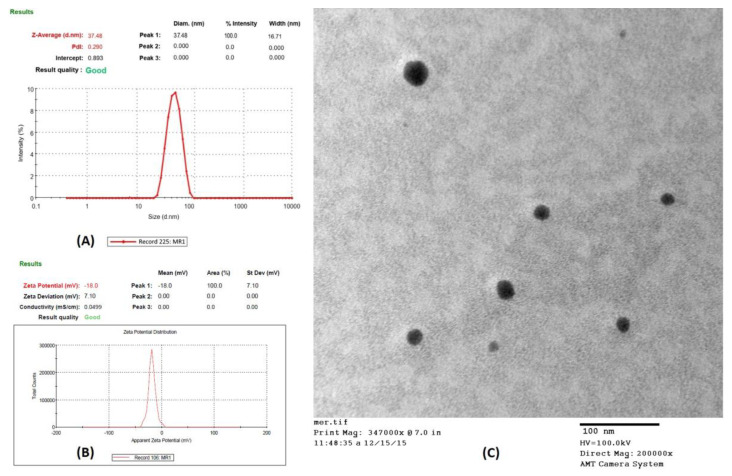
Characterization of optimized nanoemulsion (NE2): (**A**) Particle size distribution curve; (**B**) Transmission electron microscopy (TEM) analysis; (**C**) Zeta potential of NE2 formulation.

From the physical stability testing data, NE1 to NE5 were selected for further study. Several characteristics of the stable formulations (NE1-NE5) and their in vitro drug release are displayed in Table 3 and Figure 2, respectively. Accordingly, NE1 to NE5 were considered for characterization based on the results mentioned in Table 3. It was concluded that NE2 had an optimized formulation among NE1 to NE5 for further study. Additionally, as shown in Figure 3A–C, NE size was less than 100 nm based on TEM morphological evaluation, which agreed with the results of the photon correlation spectroscopy study. According to the results, the optimized formulation (NE2) had a hydrodynamic particle size of 37.48 nm and a polydispersity index (PDI) of 0.290 (Figure 3A,B). Figure 3C shows that the formulation had a zeta potential of −18 mV. NE globules with a particle size of less than 100 nm and PDI less than 0.4 are thought to be optimal for topical delivery because of the huge surface area [4,17]. The globules of NE were perfectly round, with a border that was both smooth and flexible. For the NE interfacial boundary to be stable, the zeta potential must be less than −30 mV [14].

### 2.7. Preparation of NE Gel

The results from the permeation investigations led to the selection of formulation NE2 for further mechanistic and in vivo study, and the gel was formed by adding carbopol-940 (1% *w/v*). The composition and method of preparation of the gel are described in Section 4.9. Since hydration increases the surface area, the gel matrix further reduced the penetration rate and permitted free movement of the globules within the gel [13,14]. As a result, the drug could be applied topically to the afflicted region with greater ease and for a longer period [13].

### 2.8. Ex Vivo Skin Permeation Study

**Figure 4:**
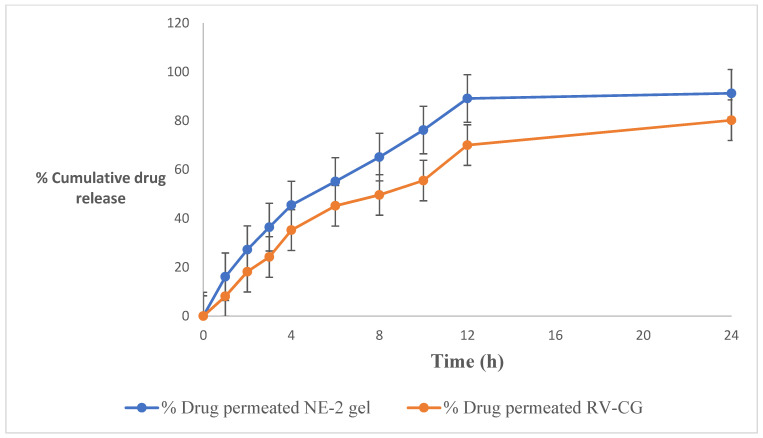
Percentage of cumulative drug release of the resveratrol-loaded NE2 gel vs. conventional gel (CG) formulation. The average value ± SD *(n* = 3) is represented by each cross bar. NE: nanoemulsion; CG: conventional gel.

Figure 4 and Table 4 depict the ex vivo skin permeation of RV-NE2 gel and RV-CG through the rat skin abdomen. A graphical depiction of the flux value was also used to assess the release behavior of the aforementioned formulation. The percentage (%) of RV that permeated from the RV-NE2 gel and RV-CG over the course of 24 h was observed in the following order: RV-NE2 gel (80.11 ± 3.12) > RV-CG (55.42 ± 2.23). The order of permeation flux (mg/cm^2^/h ± SD) of RV was as follows: RV-NE2 gel (1.23 ± 0.014) > RV-CG (0.78 ± 0.02). Permeation flow was found to be greatest for the RV-NE2 gel, followed by RV-CG hydrogel (*p* < 0.05). Permeation enhancement and a reduced stratum corneum barrier were observed in case of RV-NE2 at optimal concentrations of surfactant and cosurfactant. Additionally, RV-NE2 had a greater surface area to contact the skin and provided a stronger drug transport gradient [4,7]. Therefore, RV-NE2 gel was selected for further study.

### 2.9. Skin Drug Retention Studies

The formulations were evaluated in an experimental evaluation of medication retention. Table 4 shows the drug-retention characteristics of several RV-NE2 gels. Nearly 4.5 times more drug was retained by the NE2 gel than by RV-CG. Greater drug penetration through the skin for the NE2 formulation compared to RV-CG may lead to more medication reaching into the skin, which might improve drug delivery [4]. Results from the drug retention study indicated that the NE2 gel was the most effective delivery carrier, keeping more of the RV inside the skin for a longer period of time and thus providing more effective therapy for topical application.

### 2.10. In Vivo Studies

#### 2.10.1. Body Weight Measurements

**Figure 5:**
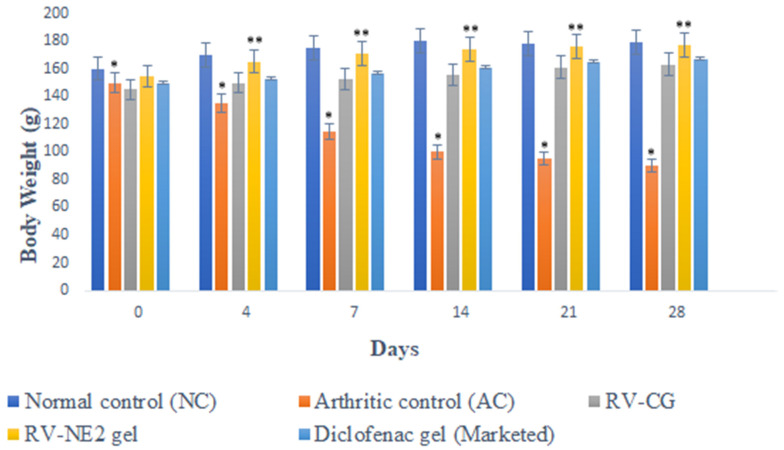
Change in body weight of different treatment groups on the days specified. The average value ± SD (*n* = 3) is represented by each cross bar. NC = normal control, AC = arthritic control, RV = resveratrol, CG = conventional gel. The comparisons were made by ANOVA followed by Tukey’s test. * *p* < 0.05 and ** *p* < 0.01 were considered significant.

After Freund’s Complete Adjuvant (FCA) injection [15], measurements of body weight significantly decreased, which correlated closely with reports of increased joint inflammation (Figure 5). In group II, there was a significant weight decrease between days 14 and 28 (*p* < 0.05). However, groups receiving therapy with RV-NE2 gel and diclofenac gel (a marketed product) did not exhibit any appreciable weight reduction after day 14 due to the impact of the drug. Moreover, once the inflammatory phase passed after day 14, the efficacy of RV-NE2 gel was noticeably stronger than that of diclofenac (*p* ˂ 0.01). Therefore, these results indicated that RV and chia seed oil via NE2 gel provided dual drug action and exerted stronger therapeutic action.

#### 2.10.2. Paw Volume (PV) Measurement

**Figure 6:**
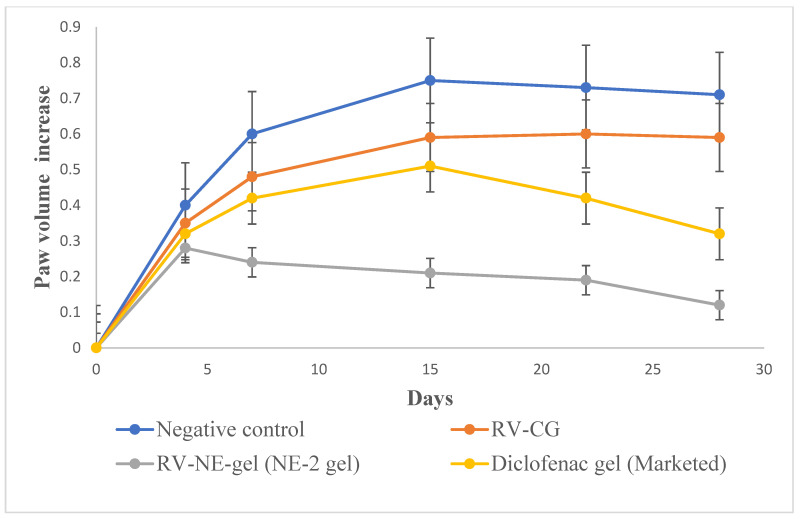
Change in in vivo parameters of different treatment groups on days specified for left hind paw volume increase. The average value ± SD (*n* = 3) is represented by each cross bar. AC: arthritic control; NE: nanoemulsion; RV: resveratrol; CG: conventional gel.

Between the first and fourth days, there were no discernible variations in PV among the groups. Beginning on day 1 and continuing through day 4, when inflammation was at its highest, an increasing trend in PV was observed for group II (Figure 6). Subsequently, inflammation began to decrease until day 14 but there was a reversal, and inflammation was also observed in the non-injected paws due to secondary lesions. The observations on the fourth, fourteenth, twenty-first, and twenty-eighth days were compared. Table 5 displays the mean AI and In% for each treatment group. When assessing the paws following arthritis induction compared with those of the normal controls, AI was used to categorize the severity of joint inflammation [15]. The AI values of the RV-NE2 gel-treated group were 55.51 ± 6.7% and 180.25 ± 6.5% on days 4 and 14, respectively, and the In% values were 75.32 ± 6.23% and 88.24 ± 5.73% on days 14 and 28, respectively (Table 5). These values were considerably higher than those of the diclofenac-treated (*p* < 0.05) and RV-CG-treated (*p* < 0.01) groups. The proliferative phase, which lasts from days 4 to 14, is characterized by inflammation, hyperplasia, and macrophage activity in the synovial membrane. RV-NE gel had a much higher rate of inhibition than RV-CG (*p* < 0.01). As a result, it was concluded that RV and chia seed oil through NE2 gel offered dual pharmacological action and contributed to increased therapeutic efficacy over RV-CG and diclofenac gel.

#### 2.10.3. Biochemical Estimation of Cytokines in Joint Tissue Homogenate

**Figure 7:**
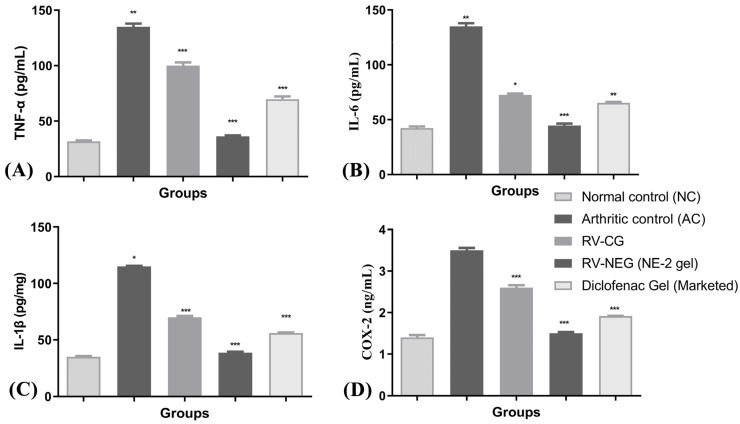
Assessment of arthritis via modulation of pro-inflammatory mediators among different arthritic treatment groups vs. arthritic control. (**A**) Level of TNF-α in joint tissue homogenate. (**B**) Level of IL-6 in joint tissue homogenate. (**C**) Level of IL-1β and (**D**) level of COX-2 in joint tissue homogenate. NC, normal control, AC = arthritic control, TNF-α = tumor necrosis factor-α, IL-6 = interleukin-6, IL-1β = interleukin 1β, COX-2 = cyclooxygenase-2. The comparisons were made by ANOVA followed by Tukey’s test. * *p* < 0.05, ** *p* < 0.01, and *** *p* < 0.001 were considered as significant.

On the 28th day after arthritis was induced, the levels of pro-inflammatory cytokines (TNF-α, IL-6, IL-1β, and COX-2) were considerably higher in group II rats than in group I (normal control) rats (*p* < 0.01). The levels of TNF-α and IL-1β were significantly lower in groups III, IV, and V. However, group IV treated with RV-NE2 gel displayed the most noticeable impact, followed by the group treated with commercial diclofenac gel (Figure 7A–D). Following their activation by bacterial lipopolysaccharides, macrophages, monocytes, neutrophils, T cells, and NK-β cells produce TNF-α. IL-1β may also encourage synovitis and speed up the deterioration of cartilage and bone [15]. TNF-α may be a critical stimulator for the production of other cytokines, such as IL-1β, in the arthritic joint. Inflammation is brought on by the affected joints’ production and release of IL-1β, IL-6, COX-2, and TNF-α, which act synergistically. TNF-α inhibits synovial mononuclear cells from the joint by spontaneously producing IL-1β [15]. Therefore, the increased levels of TNF-α, IL-6, IL-1β, and COX-2, in group II showed the severity of cartilage injury [15]. In comparison to RV-CG, the RV-NE2 gel demonstrated the most notable reductions in TNF-α, IL-6, IL-1β, and COX-2 levels (*p* ˂ 0.001), which was attributable to the increased permeability attained by the NE2 gel. Moreover, all of these pro-inflammatory cytokines are also involved in enhancing the clinical signs of arthritis and expansion of arthritic symptoms, including body weight loss and joint swelling [15]. Overall, it was concluded that RV and chia seed oil through NE2 gel offered dual pharmacological action attributed to significantly higher modulatory effects on the aforementioned pro-inflammatory cytokines.

## 3. Conclusions

In this study, a topical RV-loaded NE2 gel was developed and investigated for the treatment of inflammation caused by arthritis. According to in vitro research and ex vivo permeation and deposition study, NE as a delivery mechanism provides tremendous potential for drug transport to the target by bypassing the stratum corneum barrier. The in vivo studies demonstrated that topical treatment with RV had promise since it may effectively reduce local macrophage activity, as shown by the decline in levels of pro-inflammatory mediators (TNF-α, IL6, IL-1β, and COX-2) in the tissue homogenate. Therefore, it can be inferred that the RV-loaded chia seed oil-based nanogel has great promise for managing and controlling the development of arthritis. In the near future, biocompatibility and pharmacokinetics experiments should be conducted for the developed RV-loaded chia seed oil-based nanogel. Indeed, this study will be very useful for the improvement of RV therapeutics.

## 4. Materials and Methods

### 4.1. Materials

RV was bought from Sigma-Aldrich Co. (St. Louis, MO, USA). Hi-Media (Mumbai, India) supplied Cremophor^®^ EL, Tween 80, and PEG 400. Transcutol^®^P, Labrafac PG, and Labrasol were graciously provided by Gattefosse (Bad Krozingen, Germany). Chia seed oil was received from Nutriplanet Foods Pvt. Ltd. (Karnataka, India). Analytical grade methanol and acetonitrile were purchased from Merck Millipore (New Delhi, India). All tests were conducted with water purified by a Milli Q water purification system (EMD Millipore, Billerica, MA, USA). BASF Corporation (NJ, USA) supplied Pluronic^®^ F-127. All other chemicals used in this investigation were of analytical purity and water was double-distilled.

### 4.2. Determination of λ_max_ of RV in Ethanol

UV-VIS spectrum analysis was performed for RV, covering wavelengths from 200 to 800 nm, using a GENESYS 10S double-beam UV-visible spectrophotometer (Thermo Fisher Scientific). Each sample was preserved in a quartz cell after being diluted with 2 mL ethanol. Before placing the material in the cuvette and orienting the spectrometer in the correct manner, the device required around 20 min of calibration time [16]. To maximize absorbance, a cover was used to block out light and prevent scattering. The absorbance spectrum was determined by comparing the scanned sample’s absorbance to that of a blank sample, and the apparatus was then permitted to scan across a range of wavelengths [16].

### 4.3. Solubility Studies for Excipient Screening

Solubility studies were performed to determine the appropriate oil, surfactant, and cosurfactant concentrations [4,17]. Oil (2 mL) was mixed with a fixed quantity of RV until it became cloudy or opaque. The oils included sesame oil, triacetin, chia seed oil, castor oil, Labrafac PG, isostearyl isostearate, olive oil, and Miglyol 810N. A mechanical shaker was used to continually mix the vials at 37 ± 0.5 °C for 24 h while they were securely stoppered. In addition, after being centrifuged at 2000 rpm for 15 min, the supernatant was further filtered using a membrane filter with a 0.45 µm pore size [1]. It was then used to dilute an additional portion of the filtrate, which was then examined by UV-spectroscopy. To choose the best surfactant, various surfactants were investigated, including Cremophor^®^ EL, Tween 80, Tween 60, Tween 20, and Labrasol. Co-surfactants PEG 400, Plurol Oleique, propylene glycol, and Transcutol^®^P were also chosen. RV solubility was evaluated in the manner described above.

### 4.4. Construction of Pseudo-Ternary Phase Diagrams

The aqueous titration method was used to create pseudo-ternary phase diagrams by gradually adding water to a mixture with known proportions of oil and surfactant, then mixing the resulting liquid at 25 °C using a vortex mixer [4,17]. The ratios of surfactant to co-surfactant (S_mix_) were 1:1, 1:2, 1:3, 2:1, 3:1, and 4:1. The oil and S_mix_ were combined in a range of nine different weight ratios (1:9, 1:8, 1:7, 1:6, 1:5, 1:4, 1:3, 1:2, and 9:1). A low-viscosity, clear, isotropic, thermodynamically stable dispersion was achieved by titrating the oil and S_mix_ mixture with distilled water. This was carried out to identify the NE’s viable operating region. Then, the water volume was measured. Plotting the S_mix_ phase, water phase, and oil phase employed in the experiment allowed for constructing the pseudo-ternary phase diagrams [4,17].

### 4.5. Formulation of RV-Loaded Nanoemulsion

To create RV-loaded NE, we first dissolved RV (0.25 percent *w/v*) in chia seed oil, then gradually added water and S_mix_ (Cremophor^®^ EL and Transcutol^®^PP as surfactant and co-surfactant, respectively) while continuously stirring with a magnetic stir bar.

### 4.6. Thermodynamic Stability Testing of Nanoemulsion

Ratios of oil content between 5% and 13% and S_mix_ between 40% and 55% were chosen from the pseudo-ternary phase diagram and then subjected to an evaluation of their thermodynamic stability. Parameters such as opalescence, creaming, and phase separation were evaluated. The formulations were subjected to a 30 min centrifugation at 3000 rpm and room temperature [11]. During the heating-cooling cycle, the formulations were held at two different temperatures ranging from 4 to 48 °C for a minimum of 48 h. This exposure was run through six cycles, and stability was observed.

### 4.7. In Vitro Drug Release Study

The effectiveness of in vitro drug release of the stable formulations was evaluated using the diffusion method in a dialysis bag [4,17]. The NE was placed in a dialysis bag with a molecular weight cut-off weight of 1 kDa, and the tube was then thread-sealed. The release experiment was carried out for 12 h at 100 rpm and 37 ± 0.5 °C in ethanol/water solution (2:1) containing 0.5 g/100 mL of Tween 20 to ensure the sink condition during the experiment period. On average, 10 mL of the release medium and 10 mL NE were added to the dialysis bag (RV having a 25 mg equivalent). Additionally, the dialysis bag was submerged for 30 min in 50 mL of the release medium. At regular intervals, aliquots (0.5 mL) of the sample were removed and fresh medium was added to maintain the sink condition. UV analysis was used to determine the quantity of drug release vs. time from the samples that were collected.

### 4.8. Characterization of RV Nanoemulsion (RV-NE)

#### 4.8.1. Droplet Size, Size Distribution, and Zeta Potential

The Zetasizer Nano-ZS was used to investigate the variations in light scattering induced by the droplets’ Brownian motion and photon correlation spectroscopy was used to quantify the size and size distribution of the droplets (Malvern Instruments, Malvern, UK) [5,17]. After being appropriately diluted with water and completely mixed with vigorous shaking, 0.1 mL of the formulation was tested for light scattering at 25 °C and a 90° angle. The zeta potential of the formulation was also measured using the same tools.

#### 4.8.2. Transmission Electron Microscopy (TEM)

Transmission electron microscopy was used to analyze the droplets’ morphology [5]. The NE was diluted with distilled water (1:100), negatively stained with 2% phosphotungstic acid, and left for 30 s on a carbon-coated copper grid. After removing the surplus phosphotungstic acid using filter paper, the grid was analyzed at 60–80 kV with a Morgagni 268D transmission electron microscope (FEI Company, Hillsboro, OR, USA).

### 4.9. Preparation of RV-NE Gel and Conventional Gel (RV-CG)

The formulation that demonstrated greater permeation with less flux was chosen as the optimal formulation and transformed into a gel. Carbopol (1% *w/v*) was soaked in 3/4th quantity of water and set aside for 30 min. Then, triethanolamine (1.5% *w/v*) was added to neutralize it and the pH was adjusted to 7. Then, the remaining amount of water was added, and the mixture was stored overnight. Furthermore, Carbopol was added to the NE and mixed gently with the help of a magnetic stirrer [5]. For the comparative mechanistic and in vivo investigation, RV solution in ethanol (0.25% *w/v*) was prepared and mixed with Carbopol-940 (1% *w/v*) to produce RV-CG using the same process. Triethanolamine (1.5%) was added to neutralize the pH to 7.

### 4.10. Ex Vivo Permeation Studies

#### 4.10.1. Preparation of Skin Samples

The permeation experiments employed excized abdominal skin from Wistar rats euthanized by cervical dislocation [5,17]. A surgical blade was used to remove the animal’s skin, an electric razor was used to shave the hair from the skin, and the thickness of the skin was measured using a vernier calliper. The skin was then cleansed to eliminate any leftover fat before being washed with PBS at pH 7.4. The cleaned skin was placed in an aluminium foil wrapper and placed in a deep freezer set at −30 °C for later use [5,17]. Before concluding the in vitro skin permeation research, the skin integrity was tested using methylene blue [17]. 

#### 4.10.2. Ex Vivo Skin Permeation and Deposition Study

Franz diffusion cells were used for the in vitro skin permeation studies (Perme Gear, Inc., Hellertown, PA, USA) [17]. The cell had a 20 mL sink capacity and covered a 2 cm^2^ effective surface area when connected between the donor and receptor compartments [17]. In contrast, ethanol/water (2:1) containing 0.5 g/100 mL of Tween 20 was used as a permeation medium in the receptor compartment to maintain the sink state and a temperature of 37 ± 0.5 °C was maintained. The optimal NE formulation (RV-NE2 gel) and conventional gel (RV-CG) had 0.25% *w/v* RV. The RV-NE2 gel was applied to the donor compartment’s skin surface. At predetermined intervals (namely 1, 2, 4, 6, 8, 10, 12, and 24 h) throughout a 24-h period, an aliquot (1000 µL) was taken out of the receptor compartment and its drug content was examined using a UV spectrometric technique. The receiver volume was immediately refilled with the same volume of medium. The procedures were performed in duplicate. After the permeation investigation was completed, the skin was removed from the diffusion cell and homogenized in a tissue homogenizer for the skin deposition study [17]. The drug was then extracted using the established liquid-liquid extraction method and evaluated.

#### 4.10.3. Data Analysis of Skin Permeation

The cumulative amount of drug permeated through the unit surface area corresponded with time, so the time function t was computed and shown [4,17]. The slope of the permeation graph was used to compute the steady state rate of skin permeation, flux (Jss, g/cm^2^/h), and the intercept on the x-axis represented the lag time. The following equations were used to compute the permeability coefficient, Kp, from the flux.

#### 4.10.4. Skin Dynamics Study

The freshly prepared rat abdomen skin was placed over a Franz diffusion cell with its epidermis facing up and washed three times with phosphate-buffered saline (PBS) at pH 7.4. The donor compartment was then covered with parafilm and 1.5 g of RV-NE2 gel or RV-CG was added. Ethanol/water solution (2:1) containing 0.5 g/100 mL of Tween 20 was placed within the receptor chamber, which was then magnetically stirred continuously for 24 h.

### 4.11. In Vivo Studies

Through collaboration with T.P.S. College (Patna, India) and SS Hospital and Research Institute (Patna, India), rats (weighing between 160 and 180 g) were obtained and kept in stainless steel wire cages. A 12-h light/dark cycle and ambient temperature were maintained. The availability of food and water was unrestricted. All animal testing was performed in accordance with CPCSEA regulations (approval number 1840/PO/ReBi/S/15/CPCSEA). A total of 30 male Wistar rats were randomly assigned to five groups, each with six animals having an average body weight of 160 ± 20 g. Group I served as normal controls, group II consisted of untreated arthritis-induced negative control, and groups III, IV, and V consisted of RV-CG-, RV-NE-2 gel-, and diclofenac-treated animals. The animals were kept in propylene cages under regulated circumstances (20 ± 2 °C and 55–15%) and fed a regular pellet diet while having access to unlimited amounts of water.

#### 4.11.1. Disease Induction and Dosing Schedule

The left sub-planter paws of the animals were injected with 0.1 mL FCA [18,19]. This was prepared using 5 mg of *Mycobacterium butyricum* (Difco) pulverized with a mortar and pestle to obtain a concentration of 5 mg/mL (0.05% *w/v*) and suspended in heavy paraffin oil (Merck Millipore). Day “0” was the injection day. On the same day, twice each, the regular dosage and the drug formulation dosage were administered. This therapy lasted 28 days including the inoculation. The tibiotarsal joint was covered with a thick coating of the produced gel to fully enclose the region of the paw.

#### 4.11.2. Body Weight Measurements

The change in each animal’s body weight was determined from the day of inoculation through days 0, 4, 7, 14, 21, and 28 [18].

#### 4.11.3. Biochemical Estimation of Pro-Inflammatory Cytokines in Joint Tissue Homogenate

The experiment was ended by amputating the paw at the malleolus and cutting the digits. The skin and supporting tissues were carefully removed using a surgical blade [20]. The tibiotarsal joint was roughly sampled, and a small portion of it was carefully submerged in liquid nitrogen [21]. Using a Remi Instrument tissue homogenizer (Mumbai, India), the joint was homogenized for 30 min in 10 mL of ethanol at 15,000 rpm. The supernatant was centrifuged. Furthermore, levels of pro-inflammatory mediators, such as interleukin-1β (IL-1β), interleukin-6 (IL-6), and tumor necrosis factor-α (TNF-α), and the inflammatory mediator cyclooxygenase-2 (COX-2) were estimated using enzyme-linked immunosorbent assay kits according to the manufacturer’s instructions.

### 4.12. Statistical Analysis

The average and standard deviation of each group were used to express all the quantitative data. One-way analysis of variance (ANOVA) followed by Tukey’s test was applied to observe significant differences between the groups. *p* value less than 0.05 was considered statistically significant. All statistical tests were performed by using InStat software version 3.06 (GraphPad Software Inc., San Diego, CA, USA). 

## Figures and Tables

**Table 1:** Composition of selected nanoemulsions at the S_mix_ ratio of 3:1.

Formulation from Figure 1 Pseudo-Ternary Phase Diagrams			
Nanoemulsion Code	% of Oil (*w/w*)	% of Aqueous phase (*w/w*)	% of S_mix_ (*w/w*)
NE1	9	40	51
NE2	8	47	45
NE3	7	52	41
NE4	10	39	51
NE5	11	3	54
NE6	6	54	40
NE7	12	33	55
NE8	5	55	40
NE9	13	36	55
NE10	12	35	53

NE: nanoemulsion; S_mix_: surfactant-cosurfactant ratio; *w/w*: weight by weight.

**Table 2:** Physical stability testing of the selected nanoemulsion at the S_mix_ ratio of 3:1.

S. No.	Days/Weeks	Physical Stability		
		4 ± 2 °C	30 ± 2 °C	40 ± 2 °C
NE1-NE5	1 day	Pass	Pass	Pass
NE6-NE10		Pass	Pass	Pass
NE1-NE5	1 week	Pass	Pass	Pass
NE6-NE10		Pass	Pass	Pass
NE1-NE5	2 week	Pass	Pass	Pass
NE6-NE10		Fail	Fail	Fail
NE1-NE5	4 week	Pass	Pass	Pass
NE6-NE10		Fail	Fail	Fail
NE1-NE5	5 week	Pass	Pass	Pass
NE6-NE10		Fail	Fail	Fail
NE1-NE5	6 week	Pass	Pass	Pass
NE6-NE10		Fail	Fail	Fail

NE: nanoemulsion; S_mix_: surfactant-cosurfactant ratio.

**Table 3:** Various characteristics of stable nanoemulsions.

Nanoemulsion	Drug Content (%)	Particle size (nm)	Polydispersity Index (PDI)	Zeta Potential (mV)	% Cumulative Drug Release (Up to 12 h)
NE1	99.31	70. 21	0.321	−15.21	91.14
NE2	99.73	37.48	0.290	−18.0	98.21
NE3	99.20	100.21	0.356	−11.31	89.31
NE4	99.11	120.2	0.378	−12.34	86.12
NE5	98.92	130	0.377	−14.21	82.21

NE: Nanoemulsion; mV: millivolt; nm: nanometer.

**Table 4:** Permeation profile and drug retention of RV in skin from RV-NE2 gel and RV-CG (*n* = 3).

Nanoemulsion Code	Percentage of RV Permeated (% ± SD)	Permeation Flux (mg/cm^2^/h ± SD)	Drug Retained (mg ± SD)
NE2 gel	80.11 ± 3.12	1.23 ± 0.014	1.92 ± 0.042
RV-CG	55.42 ± 2.23	0.78 ± 0.02	0.431 ± 0.032

RV: resveratrol; CG: conventional gel: NE: nanoemulsion; SD: standard deviation; mg: milligram; cm: centimeter; h: hour.

**Table 5:** Arthritic index (AI) and rate of arthritis inhibition (In) using the volume of the hind paws of each group.

Groups	Day 4		Day 7		Day 14		Day 21		Day 28	
	%AI ± SD	%In ± SD	%AI ± SD	%In ± SD	%AI ± SD	%In ± SD	%AI ± SD	%In ± SD	%AI ± SD	%In ± SD
I	13,156 ± 12.23	*	90,120.65 ± 16.24	-	2255.12 ± 10.23	-	1380.21 ± 12.79	-	996.32 ± 13.31	-
II	0	*	0	-	0	-	0	-	0	-
III	25.74 ± 3.45	20.12 ± 7.33	80.12 ± 4.30	40.12 ± 3.17	80.21 ± 8.81	44.21 ± 5.41	81.12 ± 5.31	55.21 ± 7.15	78.72 ± 6.44	50.21 ± 5.12
IV	55.51± 6.75	36.21 ± 3.15	120.2 ± 6.12	60.21 ± 3.2	180.25 ± 6.55	75.32 ± 6.23	220.10 ± 3.11	72.81 ± 5.72	310.23 ± 6.23	88.24 ± 5.73
V	46.21 ± 3.89	32.13 ± 4.15	98.12 ± 7.21	53.24	140.51 ± 4.23	60.13 ± 4.21	170.21 ± 12.1	64.12 ± 2.39	240 ± 6.13	80.21 ± 8.72

Group I: normal control group of rats; Group II: arthritic control (AC); Group III: AC group treated with RV-CG; Group IV: AC group treated RV-loaded NE2 gel; Group V: AC group treated with diclofenac gel (marketed product); AI: arthritis index of normal animal compared with negative control; * (*p* < 0.05) compared to diclofenac gel (Group V); SD: standard deviation.

## Data Availability

The authors confirm that the data supporting the findings of this study are available within the articles and can be shared upon request.
